# A protocol for a cluster-randomized controlled trial of a self-help psycho-education programme to reduce diagnosis delay in women with breast cancer symptoms in Indonesia

**DOI:** 10.1186/s12885-017-3268-7

**Published:** 2017-04-20

**Authors:** Hari Setyowibowo, Marit Sijbrandij, Aulia Iskandarsyah, Joke A. M. Hunfeld, Sawitri S. Sadarjoen, Dharmayanti F. Badudu, Drajat R. Suardi, Jan Passchier

**Affiliations:** 1 Department of Educational Psychology, Faculty of Psychology, Universitas Padjadjaran, Bandung, Indonesia; 2 Clinical, Neuro-and Developmental Psychology, Faculty of Behavioural and Movement Sciences, VU University, Amsterdam, the Netherlands; 3 Department of Clinical Psychology, Faculty of Psychology, Universitas Padjadjaran, Bandung, Indonesia; 4 Department of Psychiatry, section Medical Psychology and Psychotherapy, Erasmus MC University Medical Center, Rotterdam, the Netherlands; 5 Department of Surgical Oncology, Hasan Sadikin Hospital, Bandung, Indonesia

**Keywords:** Breast cancer, Delay in diagnosis, Psycho-education, Self-help, Indonesia

## Abstract

**Background:**

Breast cancer (BC) is the most frequent cancer occurring in women across the world. Its mortality rate in low-middle income countries (LMICs) is higher than in high-income countries (HICs), and in Indonesia BC is the leading cause of cancer deaths among women. Delay in breast cancer diagnosis negatively impacts cancer prognosis. Only about 30% of patients who come to the hospital to check on their breast abnormalities, continue thorough examination to biopsy to get a diagnosis based on the results of anatomical pathology. Many Indonesian women with breast cancer were already in an advanced stage when starting treatment. Therefore, delay in diagnosis is a serious problem that needs to be addressed. The present study will investigate whether our newly developed self-help psycho-educational programme, “PERANTARA”, for women with breast cancer symptoms is effective to reduce patient diagnosis delay in Indonesia.

**Methods:**

A cluster-randomized controlled trial will be conducted in 106 patients in four hospitals in Bandung, West Java, Indonesia. Data will be collected at baseline (pre-assessment), 7 days after the intervention (post-assessment), and at 3 months (follow-up assessments). The primary outcome is delay in diagnosis and treatment. Secondary outcomes are breast cancer knowledge, anxiety and depression, and quality of life. Exploratively, adherence with treatment will be measured too. Data will be analysed by hierarchical linear modelling (HLM) to assess differential change over time.

**Discussion:**

If proven effective, PERANTARA will be evaluated and implemented in a diversity of settings for local cares (such as in POSYANDU, PUSKESMAS) that provide health education/psycho-education for women with breast symptoms.

**Trial registration:**

ISRCTN12570738. Date: November 19th, 2016.

**Electronic supplementary material:**

The online version of this article (doi:10.1186/s12885-017-3268-7) contains supplementary material, which is available to authorized users.

## Background

Breast cancer (BC) is the most frequent cancer occurring and the leading cause of cancer-related deaths in women, with an estimated 1.67 million new cancer cases diagnosed and 522,000 deaths in 2012 [1]. The incidence of breast cancer in LMICs is lower as compared to HICs (25.8 vs 95 per 100.000), but a major concern in LMICs is the higher mortality despite its lower incidence (12.7 vs 17.1 per 100.000) [2]. In Indonesia, BC is the most prevalent cancer among women with an incidence rate of 48,9 and a mortality rate of 19,8 women per 100,000 [3].

One of the reasons for the high mortality of breast cancer in LMICs is the fact that most cancer in LMICs is detected at later stages. Review on delay of diagnosis revealed that more than 70% of breast cancer patients in most HICs are diagnosed in Stages I and II, compared with only 20 to 50% of patients in the majority of LMICs [4]. In Indonesia, about 60%–70% of patients had their first BC-related visit to the hospital when the disease was already in advanced stages (III and IV) [5–7].

Diagnosis delay, the time between seeking medical advice and the date of final diagnosis based on pathological examination (i.e. biopsy), remains a serious problem. Studies have shown that diagnosis delay of over 3 months is associated with a bigger tumour size, positive lymph nodes, high incidence of late clinical stages, and metastatic disease [8, 9]. Delay in diagnosis predicts worse clinical outcomes [10]. The delay may cause mortality of the patient. A previous qualitative study involving 50 BC patients in Indonesia found that 35 participants (70%) were delayed in seeking help, with the time of delay ranging from 4 to 24 months, except for the four participants who had delayed for 4 years, 10 years, 3 years, and 15 years, respectively [11].

Previous studies found that delay in diagnosis among women with breast cancer symptoms was a result of a complex interaction of several factors, which often concern characteristics of the patients and the health care system. Several studies state that patients-related factors are a major concern and need to be addressed in an intervention program (e.g. lack of knowledge and awareness about breast cancer). For example, a study in Moroccan women found that diagnosis delay is also a very important health problem in Moroccan women, particulary caused by a lack of knowledge, information and awareness regarding breast cancer [12].

As diagnostic delay is a serious problem, efforts to reduce delay are necessary. Improving knowledge of women, via training and involving physicians and other healthcare providers, seems to be an effective approach to reduce delay in the diagnosis of breast cancer [13]. The delay to consult a doctor for seeking help after the discovery of the symptoms is most likely caused by lack of awareness and inadequate knowledge about symptoms and treatment of BC [11, 14, 15]. It is crucial that the information that is provided by the medical staff, is both informative and acceptable to patients.

A cross-sectional study in 70 breast cancer patients in Indonesia showed also that many breast cancer patients in Indonesia were not satisfied with the information they received [16]. The study showed that most patients were dissatisfied with the information about social support. Furthermore, the study found also that most of the patients were dissatisfied with the amount of written information. Therefore, the use of clear, concise, and easy to understand educational materials (e.g.: leaflets, posters, flipcharts table, visualized story telling) may be effective and efficient in complementing the verbal information that is usually provided by the oncologists at the oncology clinic. The utilization of educational material may be a solution to overcome the lack of resources (e.g. : experts, time and finance) and can be given by a face-to-face breast health education, either individually or in groups.

Numerous psycho-educational interventions have been designed for women (affected by) with breast cancer (or breast cancer symptoms) in response to the need for psychological support and health behavior education. A randomized controlled trial (an experimental pretest-posttest control group design) involving 76 women with BC in Turkey comparing a psycho-education program to treatment as usual reported that the psycho-educational program (which consisted of eight 90 min weekly sessions on basic information about breast cancer, nutrition, psychosocial factors, interpersonal skills, problem solving, experience sharing and support) led to positive changes in levels of adjustment to cancer [17]. In that study, the psycho-educational group had higher levels of fighting spirit, lower levels of helplessness/hopelessness, anxious preoccupation and fatalism, both at 6 weeks and 6 months after the intervention. However, there were no changes in the levels of avoidance/denial compared to the control group. Another study (one group pre-post test design) carried out in early-stage BC patients in Italy found that a psycho-educational intervention was feasible and useful to enhance coping strategies in patients [18]. An RCT that evaluated the efficacy of a psycho-educational intervention (an individualized fatigue education and support program delivered in the clinic and by phone over three 10- to 20-min sessions 1 week apart) in improving BC-related fatigue compared to general cancer educational sessions (equivalent in number and timing to the sessions that were provided for the intervention group) suggests that women who received the intervention received some short-term benefit in terms of minimization of the intensity and impact of fatigue on daily life [19]. A systematic review and meta-analysis to evaluate the effectiveness of psychosocial and especially psycho-educational support interventions for early-stage breast cancer patients showed that these interventions were effective in increasing emotional well-being [20]. An intervention study that aimed to assess the effectiveness of psycho-education on well-being status and depression among Malaysian BC patients using cluster non-randomized trial design found that group psycho-education not only significantly improved breast cancer survivors’ well-being status but also reduced depression level [21].

Web-based programs are a promising new way to offer psycho-educational interventions to patients and their caregivers [22], but several patient groups in Indonesia have no access to it, particularly patients with low education, low socio-economic status (SES) and those in rural areas. Currently in Indonesia, printed and on-line information materials about BC have been provided by hospitals, the Ministry of Health and a number of foundations or communities. However, these sources of information appeared to not have achieved an optimal impact either, due to the lack of capacity to access them, particularly for patients with low socio-economic status (SES) in rural areas. Meanwhile, the information that is accessible to the wider public (TV, radio, and newspapers) often contains promotion of traditional healing methods which even can make patients hesitant in following the process of examination (and possible treatment) at the regular hospital.

Summarizing, several educational interventions for breast cancer patients exist, but these focus mainly on coping with cancer. Interventions which focus on the reduction in diagnosis delay, are less common, and absent in Indonesia. Therefore, an educational program to diminish diagnosis delay can be of paramount importance for reducing morbidity and death. For this purpose, we developed a psycho-educational intervention (‘PERANTARA’) focused on reducing diagnosis delay.

The aim of this study is to evaluate the effectiveness of PERANTARA in reducing the delay of breast cancer diagnosis in women who visit the hospital with BC symptoms prior to formal BC diagnosis based on pathological examination. As a secondary aim, we studied its effect on breast cancer knowledge, anxiety and depression, and quality of life. Finally, we explored the effects on the longer term, also regarding the treatment delay.

## Methods

### Design

The study will be a multicentre, cluster-randomized controlled trial (cRCT), with hospitals as the unit of randomisation (clusters). We will include four hospitals in Bandung, Indonesia that have agreed to participate in this study. Because of the limited number of hospitals, we will use a cross-over design in which in each hospital will be given PERANTARA or control (treatment as usual (TAU)) at successive periods. Because every hospital forms its own control, the cross-over design can produce results with a smaller sample size than required with a parallel design. The order in which the hospital receives PERANTARA is randomly determined. The study will compare the PERANTARA to TAU in women with suspected breast cancer. This study uses two predefined periods. In the first period, two hospitals will have the experimental group (the patient is given the intervention and TAU) and two other hospitals will have the control group (patients only receive TAU). In the second period, it is the other way around.

The primary outcome is diagnosis delay in terms of the time between the date of the first consultation at the hospital and the date of final breast cancer diagnosis based on pathological examination. Secondary outcomes are breast cancer knowledge, anxiety and depression, and quality of life, as well as treatment delay.

### Participants

This study will include 106 patients in the cRCT, 53 participants in the Treatment-as-Usual (TAU) control group and 53 participants in the experiment/intervention group. Power calculations suggest a minimum sample size of 41 participants per group (power = 0.80, alpha = .05 two-sided). Taking into account 30% attrition at follow-up, at least 106 participants (53 per group) need to be included. Participants will be recruited from four district hospitals in Bandung, West Java, Indonesia, that are representative for the health care facilities in the different areas in Bandung, Indonesia.

Participants will be new patients (outpatients) who visit the hospital with breast symptoms, which make them suspects of having breast cancer. Patients eligible for the intervention program will be asked to take part in the study when reporting at the district hospitals. The following inclusion criteria will be used: women with age > 18 years, an adequate command of the Indonesian language and no major psychiatric disorder. The last is determined by checking the medical record on a consultation history/record with the Psychiatric Department. Patients who have been seen by psychiatrists are excluded from the study.

### Procedure

**Fig. 1:**
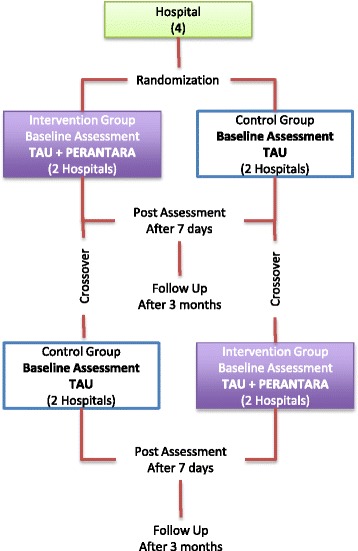
Flowchart of The Procedure

The healthcare professionals (oncologist or nurse) will inform eligible participants about the study (see Fig. 1). The research assistant will follow up to inform the patients about the details of the study and asks them for oral and written informed consent. Patients who agree to participate will be asked to fill in: (1) the socio-demographic and medical history form, (2) the World Health Organization Quality of Life - Assessment Bref (WHOQOL-BREF), (3) the European Quality of Life-5 Dimensions-5 Levels (EQ-5D-5 L), (4) the Hospital Anxiety-Depression Scale (HADS) Questionnaire, (5) the Breast Cancer Knowledge Test (BCKT). Participants who are illiterate will be giving an oral consent and a thumb print in lieu of a signature, in line with recommendations from WHO [23].

Participants in the control group will receive treatment as usual (TAU), while the intervention group will receive the PERANTARA package (which consists of printed and audiovisual materials). Participants will be requested to read and watch the intervention package within 7 days.

The post-intervention assessment will take place 7 days after the intervention. Participants will be asked to fill in the following questionnaires: (1) the Diagnostic Delay Questionnaire; Quality of Life (2) WHOQOL-Bref and (3) EQ-5D 5L; (4) HADS; (5) BCKT; (6) Intervention Material Feedback Form.

The follow up assessment will be scheduled at 3 months (12 weeks) after the post-intervention assessment. The Participant will be requested to fill in: (1) The Diagnostic Delay Questionnaire; Quality of Life (2) WHOQOL-Bref and (3) EQ-5D 5L; (4) HADS; (5) BCKT. The researcher will review the medical record of the patient to registrate the occurence of treatment delay. This period is chosen because it is also a criterion for treatment delay.

Trained research assistants with a background of psychology will conduct the assessments. All research assistants have received a 5-days workshop covering how to administer the instruments, information on common psychosocial needs of breast cancer patients, general interview techniques, and how to take a neutral role instead of a therapeutic role. Ongoing monitoring will be conducted through regular supervision by the main researcher (HS) supervised by the principle investigators.

### Intervention

The ‘PERANTARA’ is a self-help psycho-education material package that consists of both printed and audiovisual material (see Table 1). The word PERANTARA is Indonesian for ‘mediator’ or ‘facilitator’. PERANTARA is abbreviation for “Pengantar PERAwataN kesehaTan payudARA”, which means ‘introduction to breast health treatment’. The conceptual framework (blueprint) of PERANTARA was developed based on semi-structured interviews with 27 breast cancer patients and 5 focused group discussion sessions with healthcare providers (5 oncologists and 15 nurses). The conceptual framework (blueprint) was then reviewed by a panel consisting of a health psychology expert, a healthcare communication expert, an oncologist, and a breast cancer survivor. The researchers obtained suggestions and comments on the intervention blueprint and also the usability and acceptability of the developed interventions. The panel also recommended to have the printed and audiovisual material be developed by a team of audiovisual professionals. The PERANTARA provides the following information: 1) a brief explanation of breast cancer in order for the patients to have an accurate understanding and stimulation to seek information from reliable sources (oncologist); 2) an explanation of the breast examination procedure to raise the patients’ awareness on their symptoms and willingness to follow this procedure; 3) recommendation to seek support from significant persons and institutions. The audiovisual material is an 8 min and 33 s length video featuring two breast cancer survivors who shared their stories about their conditions, promoting active coping and seeking social support.

**Table 1:**
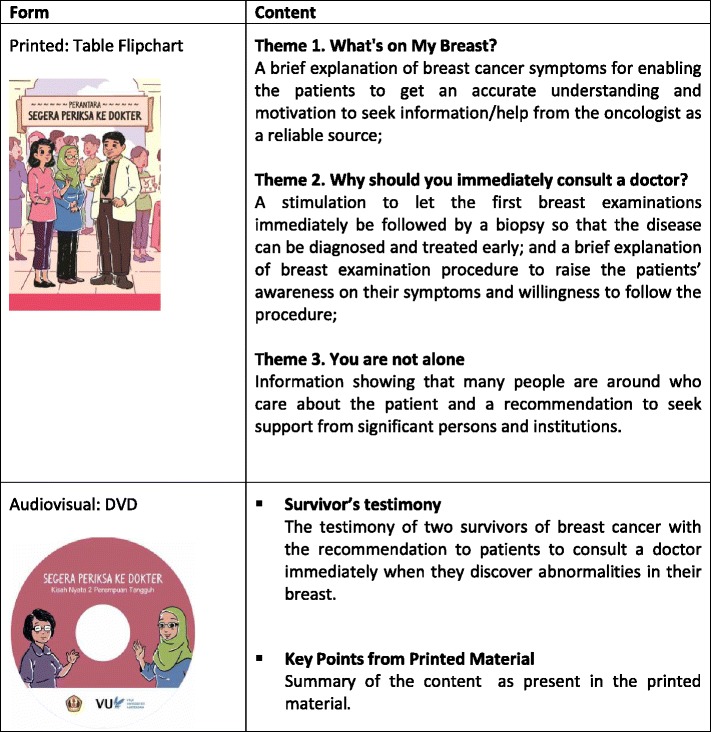
Overview of PERANTARA Package

The prototype of PERANTARA was piloted within 13 patients and with oncologists and survivors. Results from this pilot study showed that the prototype was feasible and acceptable to use. Trained research assistants will give the PERANTARA to the experimental groups. They will give a brief explanation to the patient regarding PERANTARA and instructions on how to use it.

### Treatment as usual (TAU)

TAU in a district hospital usually consists of consultation with the oncologist about the medical examination procedures. Information related to breast health is limited, and usually entails nothing more than a poster on the wall in the hospital waiting room. Psychosocial services for patients with breast complaints to support the patient during the examination process are not provided at all four hospitals participated in the study. The research assistants will keep track of all services received by the patients during the course of the study.

### Instruments

#### Primary outcome

Diagnosis delay will be the primary outcome measure. This is defined as the time between the date of the first consultation at the hospital and the date of the definite breast cancer diagnosis based on pathological examination. This outcome will be measured using the Diagnostic Delay Interviewer Guide. This instrument has been developed by the researchers and consists of the following questions: (a) What was the date that you consulted with a doctor in the hospital?, (b) What was the date that you received the breast cancer diagnosis?, and if there was a consult delay (c) What were the reasons for delay? A distinction will be made between delay due to the patient and due to the doctor. This tool will be pilot-tested before the final data collection is done.

#### Secondary outcome

A standard socio-demographic and medical history form (SDMH) will be used to collect patients’ background data on ethnicity, age, marital status, religion, education level, employment status, income level, travel time to hospital, insurance status, mental health status, family history of cancer and use of traditional healer. The patients’ medical records will also be reviewed to obtain data on type and stage of cancer and type of treatment.

Patient knowledge will be assessed using the Breast Cancer Knowledge Test (BCKT). The BCKT is a 20-item questionnaire that was developed and tested to determine (a) general knowledge of breast cancer; and (b) the relationship between the knowledge of breast cancer and utilization of screening practices. The BCKT consists of 2 subscales; one subscale of 12 items to measure general knowledge and one subscale consisting of 8 items to measure knowledge of curability. In the first section, item number 3 about the breast cancer prevalence was adjusted with the actual Indonesian prevalence rate. The total score ranged from 0 to 20, with higher scores indicating higher breast cancer knowledge. The BCKT has an acceptable reliability coefficient (α = 0.69). The Breast Cancer Knowledge Test (BCKT) has been translated into Indonesian using the forward-backward translation method [24].

Symptoms of anxiety and depression will be measured with the Hospital Anxiety and Depression Scale (HADS). The HADS is a 14-item self-report questionnaire that was developed to assess psychological distress in people with medical illness. It consists of 2 subscales: 7 items to measure anxiety (HADS-A) and 7 items to measure depressive symptoms (HADS-D). Psychometric properties of the Indonesian version of the HADS were adequate, with values of Cronbach’s coefficient (alpha) being 0.77 and 0.74, respectively [13].

Quality of life will be measured using the WHOQOL-BREF ([25]; in the Indonesian version with a 4 week-time frame). This tool is a self-report questionnaire that consists of 26 items and each item represents one facet of life that is considered to have a contribution to a person’s quality of life. Two items measure quality of life and health satisfaction in general. Twenty-four items measure four broad domains, namely physical health (7 items), psychological health (6 items), social relationships (3 items) and environmental (8 items). The WHOQOL-BREF has been validated, and indicates high validity and reliability for measuring the quality of life among the elderly [26]. We will also use the EQ-5D 5L to measure health-related quality of life [27]. EQ-5D 5L is based on a descriptive system that defines health in terms of 5 dimensions: mobility (MO), self-care (SC), usual activities (UA), pain/discomfort (PD), and anxiety/depression (AD). Each dimension has 5 levels: no problems, slight problems, moderate problems, severe problems, and extreme problems/unable. This descriptive system is then followed by a self-rating of overall health status on a visual analogue scale (EQ - VAS) ranging from 0 (“the worst health you can imagine") to 100 (“the best health you can imagine"). EQ-5D 5L has been validated in diverse patient populations in numerous countries [28]. The official Bahasa Indonesia version of the EQ-5D 5L will be used; provided by the EuroQol Group [29].

Feedback from participants on the intervention material package will be measured using The Intervention Material Feedback Form. This instrument has been developed by the researchers. Finally, treatment delay will be measured at the follow-up measurement by asking the patient with a positive diagnosis if she has started her treatment at the hospital. This will also be checked with the hospital records.

### Analysis plan

Demographic characteristics will be summarized using descriptive statistics, including percentages for categorical data, and means and standard deviations for continuous data. The descriptive statistics will be tabulated and presented graphically. Hierarchical linear modeling (HLM) will be employed to evaluate potential changes in the Diagnostic Delay Questionnaire scores over time. In this study, a three level model will be used with repeated measures nested in person, and persons nested in clusters. Time (pre, post, follow-up) will be considered as level 1, the individual persons will be considered as level 2 and the clusters (intervention-control group) will be considered as level 3. HLM analysis using a similar model will also be carried out to evaluate potential changes in the scores of secondary outcomes: Quality of Life (WHOQOL-BREF and EQ-5D 5L), HADS, and BCKT respectively. HLM analysis will be carried out separately for each phase before and after crossover. Demographic variables will be entered as additional predictors including SES, educational levels, and patients’ locations to explore potential impact on the effect of the interventions.

### Ethics

The project has been approved locally by the Health Research Ethics Committee of Dr. Hasan Sadikin General Hospital Bandung on December 23rd, 2013, Document No: LB.04.01/A05/EC/127/XII/2013 (Additional file 1).

## Discussion

Delivering self-help psycho-education materials, explicitly aimed at diagnosis delay – like our PERANTARA - may be an effective way to offer psycho-educational interventions. Therefore, this study has been designed to evaluate the effectiveness of self-help psycho-education materials in reducing the delay of breast cancer diagnosis in women who visit the hospital with BC symptoms prior to formal BC diagnosis based on pathological examination. If proven effective, PERANTARA will be evaluated and implemented in a diversity of settings of primary care (such as in PUSKESMAS, POSYANDU), which provide health education/psycho-education for women with breast symptoms who are liable to BC diagnosis delay.

## Additional file

Additional file 1: Ethical clearance approval. (PDF 626 kb)

## Abbreviations

BC: Breast cancer
BCKT: Breast cancer knowledge test
cRCT: Cluster-randomized controlled trial
EQ-5D 5L: European quality of life-5 dimensions-5 levels
EQ-VAS: European quality of life-visual analog scale
HADS: Hospital Anxiety and Depression Scale
HICs: High-income countries
HLM: Hierarchical linear modeling
LMICs: Low-middle income countries
PERANTARA: Pengantar PERAwataN kesehaTan payudARA
POSYANDU: Pos Pelayanan Terpadu
PUSKESMAS: Pusat Kesehatan Masyarakat
RCT: Randomized controlled trial
SDMH: Standard socio-demographic and medical history form
SES: Socio-economic Status
TAU: Treatment as usual
WHOQOL-BREF: World Health Organization Quality of Life – BREF
