# Supplementary material for: A protocol for a cluster-randomized controlled trial of a self-help psycho-education programme to reduce diagnosis delay in women with breast cancer symptoms in Indonesia
Source: BMC Cancer. 2017 Apr 20;17:284. doi: 10.1186/s12885-017-3268-7 (PMC5399390; doi:10.1186/s12885-017-3268-7)
Supplement: Additional file 1: — Ethical clearance approval. (PDF 626 kb) [file 12885_2017_3268_MOESM1_ESM.pdf]

KEMENTERIAN KESEHATAN RI
DIREKTORAT JENDERAL BINA UPAYA KESEHATAN
RSUP. Dr. HASAN SADIKIN BANDUNG
Jalan Pasteur No. 38, Bandung 40161
Telepon : (022) 2034953, 2034954 (hunting) Faksimile : (022) 2032216, 2032533
Surat Elektronik : humas@rshs.or.id, perjan_rshs@yahoo.com
SMS hotline : 081220050547

rshs

ETHICAL CLEARANCE APPROVAL

No : LB.04.01/A05/EC/127/XII/2013

The Health Research Ethics Committee of Dr. Hasan Sadikin General Hospital Bandung has reviewed and discussed the applied research on the meeting held on December 19, 2013. This letter is to certify that the applied research which entitle:

"Developing and Evaluating an Intervention to Address Treatment Delay and Low Treatment Adherence in Indonesian Women with Breast Cancer"

Name of the principal investigator : Prof. Dr. Sawitri Supardi Sadarjoen
Name of other investigator : 1. Aulia Iskandarsyah, Ph.D
2. Hari Setyowibowo, M.Psi

Name of Institution : Faculty of Psychology Universitas Padjadjaran
Bandung, Indonesia
Received on : December 11, 2013

Ethical clearance has been granted in Dr. Hasan Sadikin General Hospital Bandung.
Ethical clearance is valid from the date this latter has been issued until the date of the research outlined on the application form.

Please be informed the research ethics committee if there is alteration of any part of the research methodology or time of study as outline in the ethics application.

Bandung, December 23, 2013

Chairman of The Health Research Ethics Committee
of Dr. Hasan Sadikin General Hospital, Bandung, Indonesia

Dr. Hermawan Nagar Rasyid, dr.,SpOT(K).,MT(BME).,Ph.D.,FICS
NIP. 19571222 198511 1 002
